# The picture talk project: Aboriginal community input on consent for research

**DOI:** 10.1186/s12910-019-0349-y

**Published:** 2019-01-29

**Authors:** Emily FM Fitzpatrick, Gaynor Macdonald, Alexandra LC Martiniuk, June Oscar, Heather D’Antoine, Maureen Carter, Tom Lawford, Elizabeth J Elliott

**Affiliations:** 1 Discipline of Child and Adolescent Health, Sydney Medical School, the Chidren’s Hospital at Westmead, University of Sydney, Sydney, NSW Australia; 2 The Sydney Children’s Hospital Network, Sydney, NSW Australia; 3 Department of Anthropology, University of Sydney, Sydney, NSW Australia; 4 Sydney Medical School, University of Sydney, Sydney, NSW Australia; 5 The George Institute for Global Health, Sydney, NSW Australia; 6 Dalla Lana School of Public Health, University of Toronto, Toronto, Canada; 7 Menzies School of Health Research, Darwin, NT Australia; 8 Marninwarntikura Women’s Resource Centre, Fitzroy Crossing, WA Australia; 9 Nulungu Research Institute, The University of Notre Dame, Broome, Australia; 10 Nindilingarri Cultural Health Services, Fitzroy Crossing, WA Australia; 11 Kimberley Aboriginal Law and Culture Centre, Fitzroy Crossing, Australia

**Keywords:** Research, Consent, Qualitative Methods, Aboriginal, Indigenous, Community, Focus Groups, Pictures, Yarning

## Abstract

**Background:**

The consent and community engagement process for research with Indigenous communities is rarely evaluated. Research protocols are not always collaborative, inclusive or culturally respectful. If participants do not trust or understand the research, selection bias may occur in recruitment, affecting study results potentially denying participants the opportunity to provide more knowledge and greater understanding about their community. Poorly informed consent can also harm the individual participant and the community as a whole.

**Methods:**

Invited by local Aboriginal community leaders of the Fitzroy Valley, the Kimberley, Western Australia, The Picture Talk project explores the consent process for research. Focus groups of Aboriginal community members were conducted to establish preferences for methods of seeking individual consent. Transcripts were analysed through NVivo10 Qualitative software using grounded theory with inductive and deductive coding. Themes were synthesised with quotes highlighted.

**Results:**

Focus groups with Aboriginal community members (*n* = 6 focus groups of 3–7 participants) were facilitated by a Community Navigator as a cultural guide and interpreter and a researcher. Participants were recruited from all main language groups of the Fitzroy Valley – Gooniyandi, Walmajarri, Wangkatjungka, Bunuba and Nikinya. Participants were aged ≥18 years, with 5 female groups and one male group. Themes identified include: Reputation and trust is essential; The Community Navigator is key; Pictures give the words meaning – milli milli versus Pictures; Achieving consensus in circles; Signing for consent; and Research is needed in the Valley.

**Conclusion:**

Aboriginal communities of the Fitzroy Valley recommend that researchers collaborate with local leaders, develop trust and foster a good reputation in the community prior to research. Local Aboriginal researchers should be employed to provide cultural guidance throughout the research process and interpret local languages especially for elders. Pictures are preferred to written text to explain research information and most prefer to sign for consent. The Fitzroy Valley welcomes research when collaborative and for the benefit of the community. Future research could include exploring how to support young people, promote health screening and improve understanding of medical knowledge.

## Background

The Western approach to research with Indigenous communities is not aligned with Indigenous ways of knowing, doing and being [1–3]. In response, local Aboriginal leaders invited us to conduct The Picture Talk Project, based in the Fitzroy Valley, The Picture Talk Project aims to explore how to establish strong partnerships and improve the consent process for research with Aboriginal communities. The Picture Talk Project seeks practical advice directly from people living in remote Aboriginal communities of the Kimberley, Australia, asking how researchers should engage in a way that is empowering and culturally respectful [4–6].

The Picture Talk project comprises:

1. A systematic review of research publications which evaluate or describe in detail the consent process for research with Indigenous populations and an evaluation of current research guidelines [3]
2. Interviews with Aboriginal leaders about their understanding and experience with research and community consent [4]
3. Focus groups with Aboriginal community members about their research experiences and the individual consent process (reported here)
4. Feedback to the community and wider scientific audience
5. Advocacy for policy changes in current guidelines [6]

This paper will focus on part 3: findings from focus group discussions with community members.

This paper explores the question: What do Aboriginal community members of the Fitzroy Valley have to share about past experiences with research, methods for seeking consent, overcoming language and cultural differences, and suggestions for future research?

What we know:

- Few publications evaluate or describe in detail the consent process for research with Indigenous communities [3].
- A study in Alice Springs, Australia revealed that one information session was not enough for Aboriginal participants to give informed consent. Participants preferred information presented in the form of a flipchart by a doctor with an Aboriginal research officer [7].
- Bull [8] found that it was important to Canadian Aboriginal communities that researchers establish reciprocal respectful relationships, seek collective consent, provide the option of oral consent and that research be relevant to the community.
- In the USA, people of the Navajo Nation and interpreters report the consent process for research involved too much scientific and legal jargon and recommended the use of graphics to help explain research concepts in a visual way [9].
- A research team in Alberta learned it was insulting to seek consent from community elders after they had already accepted the ceremonious offering of tobacco. In response they designed a method which kept track of oral consent for research [10].
- An evaluation of international, national and local ethical research guidelines [3, 11–39] revealed that few published guidelines required that researchers provide:
- Access to an interpreter,
- Research information in the participant’s language of preference
- Visual aids for seeking consent
- Consent materials only after input from local Indigenous people.

What this paper adds:

- This is a unique first-hand account of focus group discussions sharing stories about research experiences with members of remote Aboriginal communities of Australia.
- Direct feedback about the standard consent form.
- Suggestions are made for how research information should communicated.

This paper brings to light a number of ethical issues:

- How should consent be sought for research with Indigenous communities?
- How should informed consent be evaluated and by whom?
- Does a power differential remain between the researcher and the researched?
- How important is the trusting relationship with community and the research team?

If consent for research is not obtained freely, without true understanding selection bias may occur in recruitment, affecting study results. Poorly informed consent could potentially cause harm to the individual participant or the community as a whole. By working in partnership with local Aboriginal communities, The Picture Talk Project research team worked to overcome these issues. It must be noted: The term Indigenous will be used in this paper when referring to populations around the world, while the term Aboriginal will be used when referring to the participants of The Picture Talk Project, as this is the preferred term by the people who live in the Fitzroy Valley in Western Australia [5].

## Methods

### Setting

This project is set in the very remote [40, 41] Fitzroy Valley in the Kimberley, Western Australia, with a population of approximately 4500, 80% of whom are Aboriginal [42] and belong to 4 main language groups (Gooniyandi, Wangkatjungka, Walmajarri and Bunuba) as well as Nikinya [41, 42]. Fitzroy Crossing is the main town with 45 Aboriginal communities up to 200 km away on open roads which get flooded annually in the ‘Wet Season’ [42, 43]. The Picture Talk Project was inspired by the positive impact of the Lililwan Project conducted the year prior [44–52]. The Lililwan Project was a fetal alcohol spectrum disorder prevalence study conducted in partnership with the Aboriginal communities of The Fitzroy Valley and was highlighted in the Social Justice Report by the Commissioner for Aboriginal and Torres Strait Islander people at the time [53]. The consent rate for the Lililwan Project was 95–97% [46–49]. Following this, The Picture Talk Project was invited to further explore research partnerships, the consent process and seek practical advice on how to engage Aboriginal communities in a way that is empowering and culturally respectful [4, 5].

### Collaboration

A research leadership team was formed including local Aboriginal leaders and external researchers. Community Navigators who had local respect, local knowledge and experience working between the Aboriginal and Western worlds were employed as local researchers [5]. Community Navigators were male or female and employed from all language groups in line with local cultural protocol. Community Navigators interpret language for elders and other participants and explain cultural protocols for external researchers [4, 5]. Skills for conducting qualitative research were developed through training Community Navigators. Other studies report benefits of collaborating with local Aboriginal people when conducing focus group research [54, 55] or having a steering committee or reference group [1, 56]. Following the literature review, Aboriginal community leaders were interviewed about how to approach Aboriginal communities for research as part two of The Picture Talk Project. This advice directly supported the research team’s approach to recruiting community participants for the third part involving focus groups. By using initial insights from research and applying them to support the design of subsequent stages, grounded theory was applied [57].

### Consultation and community consent

Community presentations were conducted in partnership with local Aboriginal leaders at various meetings around the Fitzroy Valley to obtain community approval and consent for The Picture Talk Project [4, 5]. Meetings included 8–40 people, for example the Fitzroy Valley Futures Forum attendees included local community representatives as well as local and visiting service representatives from government and non-government organisations [58]. Other presentations were given directly to key organisations as approved by their CEO or to families who wanted to know more about the project. The Picture Talk Project was named by Marmingee Hand who is a local Aboriginal leader, after the locally adapted process of using pictures to help explain the Lililwan Project consent forms. A logo was designed with local artists Neil Carter and Community Navigator Sandra Nugget to represent the spirit of the project [5]. This was used as visual identity on research uniforms, posters, car magnets, presentations and information sheets/consent forms [5]. The project was advertised through posters that included the project logo, pictures of the research team and logos of supporting organisations, namely Nindilingarri Cultural Health Services, Marninwarntikura Women’s Resource Centre, Kimberley Aboriginal Law and Culture Centre and the University of Sydney. These were placed in key parts of town and in the windows of the project car.

### Participant recruitment and consent

Aboriginal community members aged ≥18 years were recruited to Focus Groups through passive snowball sampling [59–61] as recommended by the local Aboriginal leaders on the research leadership team. They were invited to approach the research team if they were interested in participating. Individual consent was sought from participants by a researcher in the presence of a Community Navigator. Working closely with a Community Navigator during each stage of the research process had been highlighted as an essential part of culturally respectful research in The Picture Talk Project interviews with Aboriginal community leaders [4].

The aim was to recruit a representative sample of the population and collect data until there was saturation of topics [5, 57, 59–66]. A participant information statement and consent form written in plain English were explained in detail prior to commencement of the focus group. After any questions were answered, signed or verbal consent was obtained (witnessed by a Community Navigator). Participants then provided demographic information including their language group, preferred language, age group, education level and cultural knowledge. The last category was included as not all participants had completed mainstream schooling but were nevertheless valued amongst community members as a source of cultural knowledge. Showing respect for the community’s traditional knowledge and local expertise was highlighted as important during interviews with Aboriginal community leaders and this respect was also applied to focus groups [4].

### Focus group stratification and questions

Since many of the Aboriginal community members had limited experience with participating in research projects, Aboriginal leaders on the research team advised that it was preferable that participants be invited to participate in a focus group discussion rather than a one to one interview. Focus group structure and questions were informed by qualitative research and Aboriginal leaders on the research team and were guided by participant interactions [5, 60, 61, 63, 67–71]. Questions were focussed on experiences with research and the consent process [5]. The discussion was adapted to be delivered in a way that was akin to Yarning, which is a way of communicating in a story-teller format [5, 68, 69, 72]. Rapport is established through “social yarning” then the discussion is formalised into “research topic yarning” [68]. With consent, focus groups were recorded and responses transcribed verbatim for analysis. Questions were asked by the researcher and immediately interpreted and explained in local language or Kimberly Kriol by the Community Navigator. Having a Community Navigator present to interpret or explain things was also mentioned as important during interviews with Aboriginal community leaders [4].

Focus group participants discussed [5]:

- Understanding of and past experiences with research
- Sometimes participants wanted to discuss participation in research with a particular family member or health worker rather than the Community Navigator
- Whether having a Community Navigator present was helpful
- How to receive information about research and preferred place to conduct research
- An excerpt from the participant information statement of the Lililwan Project typed in plain English was compared to a flip chart of either photographs or cartoons
- The use of stories in describing research concepts
- Suggestions for future research

Participants could nominate the time and the place of the focus group. During interviews with Aboriginal community leaders, it was emphasised that Aboriginal people really valued opportunities to work ‘on country’ [4]. ‘On country’ was a term used locally to describe an area which was linked to a group’s ancestral history and holds cultural and spiritual significance [73, 74]. Lunch was provided in gratitude and participants helped in its preparation. Participants were invited to draw during discussions. EF joined in this activity to encourage a sense of collaboration/partnership. Having a mutual activity took performance pressure off participants as they unpacked their ideas.

### Analysis

Focus Group sessions either recorded or documented with consent, and transcribed to Microsoft Word documents then uploaded to NVivo 10 qualitative software [5, 65, 75]. Data were analysed using grounded theory through inductive and deductive coding line by line in an iterative process [57]. Initial codes were deduced at the end of the focus groups reflection with EF and the Community Navigator [71]. The remaining codes were derived through inductive coding by listening and transcribing the recordings and later by reading transcripts of the focus groups using NVivo10 qualitative software [65, 75]. When working with NVivo, codes such as “Written consent” were called ‘nodes’. Many nodes were derived from the data and similar nodes (minor nodes) were grouped into major node categories [65, 75]. A node hierarchy was created in this process and key themes were synthesised [5, 65]. These themes were representative of the overarching values provided by focus group participants.

### Ethics

In addition to local cultural protocol, this project also upholds the six main values depicted in the National Health and Medical Research Council guidelines for conducting research with Aboriginal communities [11]. These are Respect, Reciprocity, Equality, Survival and Protection, Spirit and Integrity and Responsibility. The Picture Talk Project is reported in line with the COREQ guidelines for qualitative research [76].

This project was approved by the University of Sydney Human Research Ethics Committee (No.2012/348, reference14760), the Western Australian Aboriginal Health Ethics Committee, the Western Australian Country Health Service Research Ethics Committee (No.2012:15) and the Kimberly Aboriginal Health Planning Forum Research Subcommittee (No. 2012–008).

## Results

In keeping with the passive snowball recruitment design community members who were interested in participating approached the researchers. The diversity of the participants was approved and acknowledged as culturally appropriate by community leaders, and data were collected and coded until themes were saturated and no new codes arose [5, 59–66]. A total of twenty-six participants were recruited. Table 1 demonstrates the age, sex, language group and education of participants.

**Table 1:** Demographics of focus group participants

Participant Demographics		No.	%
Sex	Female	19	73
Identity	Aboriginal	26	100
Age	21–30	9	35
	31–40	5	19
	41–50	6	23
	51–60	1	4
	61+	5	19
Language Group	Walmajarri/ Wangkatjungka	12	46
	Gooniyandi	8	31
	Bunuba	5	19
	Other	3	12
	Nykina	1	4
Preferred Language	Kriol	15	58
	Walmajarri	7	27
	Aboriginal English	5	19
	Gooniyandi	3	12
	Standard Australian English	2	8
	Bunuba	1	4
Cultural Knowledge	Parent/Grandparent	11	43
	Elder	5	19
	Cultural Advisor	4	15
	Art/ music	3	12
	Interpreter	1	4
Education	Training Courses	12	46
	High school	9	35
	Did not say	3	12
	Primary	2	8
	University	1	4

Three participants declined to continue in the study due to sudden news of a death in the family and out of respect for “sorry business” (cultural mourning) they were not approached again. This was in-line with advice provided during interviews with Aboriginal community leaders – one must leave families alone if they have sorry business [4]. Hence the participation rate of those volunteering was 88%. Participants ranged in age from 18 to 70+ years and were from all main language groups of the Fitzroy Valley. Participants from other language groups (*n* = 3) were potentially identifiable so are not specified. Participants came from communities in Fitzroy Crossing town and more remote communities of the Fitzroy Valley.

Six focus groups were formed with three to seven participants per group. There was one large male group and five female groups. Focus groups comprised family units, colleagues from a local organisation, and people from the same language group and/or the same community. Three focus groups were conducted in an office with tables and chairs. Two groups were conducted sitting on chairs out the front of a community house while participants supervised their children playing nearby. One group was conducted on the banks of a river while participants fished on country [73, 74]. Focus groups comprising members of an organisations were conducted in the work place, while focus groups of community members from a particular language group were to conducted on country or in the community in which they live. Having a mutual activity such as preparing lunch together before the official start of the research helped facilitate “social yarning” [68]. In addition, focus groups that used drawing sometimes wrote down thoughts rather than bringing them up verbally in the group. Others drew small figures or pictures of country. These drawings were akin to doodling [77, 78].

### Key themes

A total of 6 themes were synthesised from the focus group data. Participants identified that for research: Reputation and trust is essential; The Community Navigator is key; Pictures give words meaning – Milli milli versus pictures [79]; Achieving consensus in circles; Signing for consent; and Research is needed in the Valley.

#### Reputation and trust is essential

Participants recognised the logos of organisations supporting The Picture Talk Project on posters [5]. Participants explained that the organisations such as Marninwarntikura Women’s Resource Centre had a good reputation in the communities and were trusted as working with the community’s interest at heart. Having Marninwarntikura logo on the research posters, symbolising that leaders of this organisation supported The Picture Talk Project, helped with participant recruitment and seeking consent [5].

During focus group sessions, participants were shown an excerpt from the Lililwan Project participant information statement which included the Lililwan logo at the top of a written explanation of the project in Plain English [5]. This is a picture of a baby surrounded by different coloured circles which represented the family and community’s responsibility for the health of each child in Fitzroy. Discussions indicted that the logo and the reputation of the Lililwan project held more value, than the text.

Focus Group 1, Participant 1 (FG1P1)*: People get confused... But you have a good thing there,* (pointing to the Lililwan Project logo)… *If they look then they are like ah Lililwan…it’s good that you’ve got simple words to say what the project’s all about.*

There was consensus around the group about this issue. Participants would smile and eyes soften in recognition of the Lililwan Project logo. The body language seemed to convey a positive attitude through the excited manner in which they talked about this project and the high tone of voice.

FG1P1: *Especially for young Mums, it’s good for them* (referring to the presence of the Lililwan Project Logo)…*They like looking at pictures, they don’t like reading… You got a good logo there with the baby.*

FG1P3: *If they’re just looking at paper, they don’t want to know, they don’t want to know about it.*

By repeating the phase “they don’t want to know”, with a low, dismissive tone of voice, the participant makes special emphasis on the point that the young mothers of the community were not likely to be engaged (and hence participate) in a research project if the information they were given was only in the format of a page of typed plain English. By adding the smallest detail such as a Logo, the page held a whole new meaning.

#### The community navigator is key

In addition, the reputation and respect that was held by the Community Navigator working with the project made a significant impact on the recruitment rate of participants.

FG1P1: *But if you’ve got a Community Navigator, you know working alongside of you, then you can give the paper work to everybody.*

Participants emphasised that it was important for the Community Navigator to be the one to initiate contact with potential participants.

FG4P2: *As soon as you jump in the car to go see the school or a parent or thing, you let that Community Navigator take the lead.*

Participants insisted that before a Community Navigator commences a research project, they need to be properly trained and have a good understanding of the project before they approach others with the information.

FG4P2: *Before you educate our people you got to educate the Navigators to do the talking.*

Having a local Community Navigator there as part of the project also meant that people had the option to hear the information in Kimberley Kriol or their own language as well as have the research content broken down into relatable concepts. It was especially important to have someone who was available to interpret language and content when communicating with the elders of the community.

FG1P1: *The old people need an interpreter…Depends if you have good English, yes you can speak...But if there’s old people, I think they need interpreters. Some don’t know English very well.*

English is often a third language to the elders of remote Aboriginal communities.

#### Pictures give the words meaning – Milli milli versus pictures

When asked about the Milli milli (written text) [79] in the excerpt from the participant information statement for the Lililwan Project, participants pointed out a number of issues including literacy in the community. Literacy is dependent on educational opportunities and many adults never had a formal education (Table 1).

FG1P2: *Some of them can’t read.*

FG4P2: *No. Not many here in the community are going to understand that* (referring to the typed text)*.*

FG4P5: *Must be a two-way sort of thing. Make it a bit simpler too for some people to understand.*

‘Two-way’ is a term used locally when referring to concepts explained from both the Aboriginal and Western world views. In this particular instance, the participant is indirectly pointing out that the information sheet (which is simply adhering to ethics committee standards) is too Westernised. The content is not simplified enough for people whose first language may not be English and do not come from a scientific or legal background. Community members suggested that information could be written in Kimberley Kriol and presented with pictures. Kimberley Kriol is the common language of the area. This community use visual means of communicating much more than the written word.

FG2P1: *Yeah I reckon in Kriol. But put some pictures in it too.*

Focus group participants were shown samples of the flipchart used for seeking consent for the Lililwan Project which included photographs of local children demonstrating how they would participate in the various assessments including one photo of a child being examined by the doctor with his carer present [5]. Simple text was put with the photographs to help explain the activity.

Some participants thought it was a great idea to have pictures to be used in conjunction with the written text to make it easier to understand, especially for those who could not read or speak English as their first language.

FG6P4: *When you got pictures it explains better than words. Telling them what the nurse doing and everything and the actual picture is showing what they’re doing. That explains to other people quicker instead of just having long words. If you have something like that it sort of sinks in quicker and people understand quicker than reading. They got to read it about 3 or 4 times before they understand the writing.*

FG1P1: *If you have local photos, people photos, you know they’ll feel proud and they’ll build up the courage to* b*e checked up.*

FG5P1: *See these people, all the old people can tell by the pictures so they can talk for the little one. They can tell from the picture what’s really happening there.*

Other participants did not like the idea of local people being used in the photos as it may cause jealousy amongst the community.

*FG1P1: What FG1P2 is saying: Sometimes people make comments you know they’ll say ‘Why does it have to be that family?’, ‘Why does it have to be that particular photo of them mob?’, you know some people get a bit. You know some people get offended when they see things more of the other side. There’s always competition.*

This participant has given indirect feedback that in order to minimise the chances of “jealousy” or bias, it would be good to include a variety pictures of people from all of the different language groups.

Focus groups raised the issue of consent, they emphasised that the people in the photograph needed to give permission to researchers to have their photograph taken for the research project.

FG1P1: *Well for me I reckon, see when the boys get older they’ll feel shame or shy. Maybe that might come along later on… You can check with them first. You know ask Mrs X and the boys, would you like to be part of it? You know in the hospital reception area for people to read or look.*

Focus group participants also raised the cultural issue that people who have died should not be shown in a photograph especially to those who knew them.

FG6P1: *Maybe if someone passed away. There could be a problem. People might get offended by it.*

They suggested a solution to this issue maybe to warn people at the start with a disclaimer that the content may contain photographs of local Aboriginal people that have died:

FG6P2: *Maybe you could write up on the front that there is this person in the photos.*

This type of warning is often used in public forums and in Australian media when showing films or photographs of Aboriginal Australian people from the past [80].

The photographs used in the Lililwan Project were converted to a cartoon using Adobe Photoshop CS6 [5]. The cartoon version was presented to the focus group participants as an alternative to the photograph they were asked about their thoughts and preference. Some preferred the cartoon version.

*FG3P2: I think cartoon is better because pictures say the person has passed away. It’s a reminder looking at the pictures especially little kids you know. Say if you show that to a parent and they see it, I think cartoons is the best way.*

*FG5P1: You look here, see small one standing out than this with the line* (referring to the cartoon). *It’s a little bit darker there* (referring to the photo)*. Clearer yeah and brighter. You can see the colours.*

By using simple computer graphics to capture the images that were in the photographs, the image was made much simpler and easier to see. In addition, subjects can be de-identified so the local child does not have to run the risk of “feeling shame” [81] and also removes the cultural issue of showing photographs of people that have passed away.

Following this, focus groups were asked about the use of story to explain research information. For example, the story of how to prepare a cycad nut for eating could be used as an example of how to eradicate strongyloides and scabies through hygiene and using medicine [82–84]. Participants agreed that stories are another good way to introduce an idea to the community. One participant answers this question with her own story, explaining how she had to interpret to her female elders the importance of pap smears.

FG1P1: *I had to tell them in a different way, in a funny way, in a cultural way* (private women’s business)*…was a good thing for the woman of the family they have to go through pap-smears. We never used to have pap-smears that in the early days. That’s why a lot of women have died through from having cancer of the cervix. I was part of this, because I was the liaison officer at the hospital.*

Others felt that the story should be presented together with the pictures.

FG2P4: *Another good way yeah you do a picture and you do a story.*

#### Achieving consensus in circles

Participants often achieved consensus about a certain issue by talking in circles within each focus group. This appeared to came about through one person starting to make a statement about a topic, then the next person repeating part of the last few words in agreement and adding it their point of view or knowledge on the subject. The last part of their sentence might then be echoed by the first person or another until silence occurred. Those who did not agree would then have space to speak at this point. Sometimes the conversation flowed as if participants were exploring an idea first, with the pros and cons, before making up their mind on a matter. At other times questions were raised but consensus was not achieved. This process of discussion, debate and decision as a group was repeated with almost every topic raised by the research team. It almost had a rhythm to it with some participants repeating parts of sentences like an echo and nodding together. Even the focus group facilitator EF found herself doing the same where it seemed natural to do so to encourage discussion. This intricate process was like the researchers and focus group participants were sharing a story together, coming to an understanding and knowing together.

#### Signing for consent

When asked if participants wanted to give verbal consent or sign consent for research projects, most community members preferred to have the agreement in writing.

FG6P1: *Probably in the long run better to be on paper just in case.*

However, there was some confusion as to why participants sign their name. Some had the expectation that they would be given money for signing a form, suggesting that they did not understand the information on the form, or the explanation of the research process and brought to light assumptions participants had when presented with paperwork.

FG1P1: *See they think every time they sign they get paid for it.* (giggling)

Before participants would grant consent for certain research projects, it was important for certain family members to be consulted as part of cultural protocol.

FG6P1: *Well a lot of people they ask their uncle or aunties who are older for their advice and their permission.*

If they decline consent they will often just avoid the research team:

FG1P1: *Sometimes you’ll see people who don’t want to be part of a project, you’ll see they’ll walk away.*

Participants were also asked how much information they would want to know before starting a project. Some thought it was good to know all the fine points of the project:

FG4P2: *You’d want to know everything!*

Others specified that the information needed to be presented over number of sessions, starting with a simple framework to build on.

FG6P1: *Probably just the basics really. Probably just have like an induction and if they want more information they can find out. Just get the basics and if they want to know more you could make a video about it.*

FG4P5: *Just not in one day but a couple of days.*

FG4P2: *For a couple of hours you’re not going to have all the information, we won’t know everything in a couple of hours.*

FG4P5: *You could run a workshop of something like that.*

Participants were full of ideas and suggestions as to how to present information so that informed consent could be obtained. It was made clear that a single presentation about a research project when seeking consent for participation was not enough.

#### Research is needed in the valley

The focus group participants were asked about their understanding of and experience with research. Some demonstrated a very clear understanding.

FG1P1: *Research is like for finding out things. You know there’s something there that’s not right. Like mothers were drinking say for example the Lililwan project. Sometimes mothers don’t know they are harming their babies cause sometimes they just drink because they like to drink, but they don’t know it’s gonna to harm their baby or something. That’s why maybe kids grew up and have behaviour problem, that’s why kids at school, kids growing up with that problem ‘cause they had a long time acting up*

Participants commented on research of the past which was sometimes conducted without informed consent.

FG2P1: *The research is to find out the things. People what to know about that thing. In the past people didn’t explain what they were doing.*

FG3P1: *Yep and I think this is the only area that has improved and doctors are explaining after the blood tests and everything. Before they never used to they just get a blood test or they’d do your finger and they won’t say what it’s for.*

Focus groups participants reported that it was not always clear when health care and research were being conducted simultaneously.

FG1P1: *Sometimes they’ll understand what doctors are talking about, but they don’t know what the research is.*

For some focus group participants, The Picture Talk Project was the first time they were (knowingly) part of a research project.

FG5P1: *I don’t really know what it is really. It’s the first time for all of us. You’re the first lady.* (Giggling) …*That’s why we don’t really know what the research is all about.*

Others had some stories to share about projects they were part of.

FG1P1: (Interpreting FG1P2) *To her, she said when we did the research with the TRACK* [85]*, that was a couple of years ago,* …*it was good to put out there, you know what the scientist say. Talk about the fish and the river, that’s important.*

When asked what future research the Fitzroy Valley communities need, participants discussed a number of topics. Some participants were overwhelmed by this question:

FG4P2: *We’re facing all sorts of issues here. All sorts of problems.*

Some participants were concerned about young people and recommended that research focus on how to support their mental health and prevent alcohol, smoking and drug use in the community.

FG2P3: *We had a hard time with them sniffing. She said I don’t want to follow them because they will want me to sniff. They will force me to sniff…Show them what happens with smoking. She’s trying to smoke cigarettes.*

Focus group participants also suggested researchers should look into how to support the financial stress and mental health of young mothers in the community:

FG3P1: *Some are single mothers who are really struggling. I think they need some sort of support. The should do a research on all areas – financial, medical…or mental stress.*

Another participant wanted research investigating whether the incidence of lung cancer in people of The Fitzroy Valley was attributable to the asbestos in their houses.

FG1P1: *Well there’s a lot of things, you know like, we have people that have cancer especially of the lungs. You know like they have, there’s a big thing about asbestos* [86, 87]*. See a lot of our mob have lived in asbestos houses and now it’s like a wake-up call for people to look into it because we may have lost a lot of people to asbestos, you know with cancer of the lungs.*

Some participants did not differentiate between the concepts of ‘research question’ and ‘health service’ and proceeded to offer practical advice as to how the community could be supported:

*including building a youth centre, increased health screening for children and improving health literacy.*

## Discussion

Focus group discussions in The Picture Talk Project uncovered a number of different issues with regard to how to improve the consent process for research with Aboriginal communities.

Key findings from the 6 focus groups of Aboriginal community members of the Fitzroy Valley are demonstrated through the following themes:

1. *Reputation and trust is essential:* If an organisation that is known and trusted by the community is seen to be supporting a project, the research is thought to be more trustworthy;
2. *The Community Navigator is Key*: Participations preferred research information to be delivered by a local person that was respected in the community;
3. *Pictures give the words meaning – Milli mill* versus *pictures*: Aboriginal community members of the Fitzroy Valley are more visual, hence pictures and stories that are used to help explain consent material are essential;
4. *Achieving consensus in circles:* Aboriginal people of the Valley are community focussed in their way of thinking hence it was natural for focus groups to reach consensus;
5. *Signing for consent:* most people wanted to sign their name when giving consent to participate in a project, preferably with a witness as a record of what happened;
6. *Research is needed in the Valley:* The community care about the next generation and are invested in their future, more research is needed in the Valley.

The Picture Talk Project focus groups reported that if organisations with a good *reputation* were seen to be supporting a project, then the research was more likely to be *trusted*. The Lililwan Project logo at the top of the excerpt from the participant information statement was received positively in all focus groups [5, 44]. Trusting, respectful relationships had been established by researchers who worked with the Lililwan Project [44, 46]. This suggests the way that the community operates is through relatedness, which is an essential part of culturally appropriate Indigenous research methodology as described by Indigenous researchers from Australia and other parts of the world [1, 2, 8, 68, 72] Non-Aboriginal anthropologists have also documented their observations on how kinship ties and connection to country impact Indigenous social connections [88, 89]. Bessarab notes that this is why ‘social yarning’ should precede the formal research discussion [68]. More value is placed in relationships fostered over time than information provided in a moment [72, 83]. In contrast when a project does not invest time into establishing good working relationships with an Indigenous community, a different outcome can occur. Walsh-Buhi reports [90] during a semi-structured interview about a substance abuse prevention program with American Indian and Alaskan Native populations, one participant noted:

“*please don’t just hang a feather on a program or put a medicine wheel on your logo and think ‘oh well this will work*”.

It is imperative to foster genuine partnerships for successful community engagement [2–4, 8, 44, 46, 83, 91].

The Community Navigator is essential in facilitating both community engagement and the individual consent process. Each Community Navigator should work with the group that they feel comfortable to work with through kinship ties – either directly through family ties or culturally acknowledged connections for example through a person’s skin name [42]. In addition, it was against cultural protocol for a stranger to directly approach an elder without being introduced. Aboriginal community leaders of the Fitzroy Valley stressed the importance of having a Community Navigator at every stage of a project:

*“He’s like a key – he is opening the door for you. He helps connect you in the right way. He helps explain why you are here and what you want to do.* (Participant 7)*”* [4].

Russell et al. presents similar findings when interviewing participants in Alice Springs, NT, Australia about preferences for how the consent process is conducted [7]. The Aboriginal participants preferred research information presented by a doctor with an Aboriginal researcher present. In addition, a number of international, national and local research guidelines recommend having an interpreter available when seeking consent for research with Indigenous populations [12, 20, 21, 23, 30, 33–35, 39, 83, 92, 93].

Focus group participants of The Picture Talk Project preferred information to be presented in pictures rather than text or what is known locally as “Milli milli” [79]. These findings are similar with the study conducted by Russell et al. where Aboriginal participants preferred research information to be presented in flip charts with visual content over a written booklet [7]. Similar advice was recommended by medical interpreters from the Navajo nation, USA suggesting researchers use graphics to illustrate concepts and assist understanding of the consent process for a diabetes project [9].

When comparing the use of photographs versus cartoons, it is important to consider that there is a cultural sensitivity around showing photographs of the deceased [94]. Pictures have been used by other research projects, for example drawings were created by Aboriginal artists were used to depict an Aboriginal story used for seeking consent for a project with the Yolngu people [82–84]. Schoen et al. [56] describe a project based in Western Australia working with Aboriginal participants in focus groups evaluating educational resources to be used to promote awareness about diabetic foot care. Photographs of diabetic feet were preferred to cartoons as they appeared more realistic. In contrast, evaluation of preference and understanding of consent materials for research with 22 livestock workers based in Tanzania comparing text with photographs and cartoons [95] demonstrated that cartoons scored the highest for comprehension and engagement of information with participants [95]. The process of seeking consent varies greatly across different cultures [96]. These similarities may perhaps not be easily translatable.

Participants liked the idea of using stories in explaining research. Andrews et al. reports using a traditional story about cultural practices with preparing the cycad nut as an analogy to explain how to manage disease with hygiene and medication [82–84]. Wilson is an Opaskwayak Cree, from northern Manitoba in Canada and he describes in his book “Research is Ceremony” the importance of story for his people as way of explaining ideas and sharing knowledge [2]. Tafoya (Taos Pueblo and Warm Springs Indian) also emphasizes the importance stories in the Native American ways of knowing [97].

Tofoya describes how stories are told in circles in Native American culture [97]. A similar thing was witnessed during the focus group discussions from which the theme “Achieving consensus in circles” was derived. They hold a different dynamic to interviews in that responses of participants are witnessed and affects the direction of a discussion and flow of ideas and can reveal a wider variety of opinions, values and beliefs of a community [98]. This process is akin to “collaborative yarning” described by Bessarab who is an Aboriginal researcher who identifies as Bardi/ Yjindjabandi from the West Kimberley [68]. Community consensus was also described in other focus group research in Aboriginal communities based in South Australia [99]. This concept is also similar to “garma” which is a Yolnu word meaning: identifying and respecting difference, while collaborating and building agreed ways of knowing in order to move forward together [100, 101]. This particular theme highlights to Western Researchers a key difference in ways of understanding through an Indigenous knowledge framework. By ‘going with the flow’ of conversation in the focus groups, deeper discussions were able to be held and the same stories were provided with new insights.

Despite there being a preference for information to be presented in a visual or auditory way, most participants felt it was important to sign consent on paper. It was discovered that there was a misconception that money would be given after signing a form by some participants. This assumption was also made by livestock workers in Tanzania when participating in a research project. In response to feedback, the “payment” of a health check for their cattle and not money was depicted in a cartoon developed by the researchers [95]. On the other hand, monetary compensation is sometimes provided to research participants for their time and travel expenses but this practice varies greatly and is reviewed carefully by ethics committees [102]. This highlights the need for researchers to design projects together with the communities so that information is delivered in a way that is fully understood. In addition, due to the potential power differential between community leaders and community members, it would be important for researchers to clarify to participants that even though community consent has been granted, that individuals are within their rights to decline participation [103]. Researchers should then evaluate understanding of consent information with participants in order to ensure truly free, prior and informed consent to the best of their abilities [11, 83]. As demonstrated in our international systematic review, this is rarely reported [3].

Participants of The Picture Talk Project reported that some researchers did not engage with the community or even seek consent, which is similar to research with other Indigenous populations around the world [104, 105] or other populations from low-and middle-income countries [106]. In contrast, projects like the Lililwan project have been well received by the Aboriginal communities:

*“There’s only one research project that I think we’ve benefited from and that’s the Lililwan project*” (Participant 17), [4].

It was not referred to as a visiting project, it was referred to with ownership by the community. As mentioned previously, this project was highlighted as an example of good research by Gooda, in the 2010 Social Justice Report [53]. Projects which invest time in forming partnerships and addressing community priorities and collaborating with local people were much better received by Indigenous communities [3, 4, 6, 81, 83, 107, 108].

Focus group participants believed there would be the added benefit of a health check while participating in a research project. Therapeutic misconception is prevalent in many research areas as described in a review of such studies by Thong et al. [109]. Participants of a lower education level and poor insight to their condition are more likely to mistake research with clinical treatment [109–111].

When asked about suggestions for research in the future, some participants raised issues which they felt needed more research such as if there is a link between local lung cancer prevalence and asbestos in the houses of the community [86, 87]. Other participants were concerned about the mental health, smoking, alcohol and drug use of the young people in the community or how to best support the financial and mental stress that young mothers were going through. These topics were raised as issues in the community that they felt needed research however no specific research questions were formulated at the time. In some focus group discussions participants would join the conversation and offer how improved service provision to the community could address issues that were raised. Practical solutions were then explored as to how best support the community. Participants wanted their children’s health checked, especially focusing on ear health and nutrition. Ear health has been identified in the Lililwan Project as a significant issue with children from remote Aboriginal communities [47]. Health education was raised as a community priority. Participants wanted the community to be educated about diabetes and fetal alcohol spectrum disorder as well as the effects of drugs, smoking and alcohol on the body [47, 112]. Alcohol in particular has had a large negative impact on the communities of the Fitzroy Valley. In response, local Aboriginal leaders lobbied for alcohol restriction laws to be put in place and invited researchers to join in the Marulu strategy which lead to the Lililwan Project [45]. Participants voiced that there needed to be more support for young mothers. It was indirectly expressed that the women feel isolated and unsupported when raising their children, this would be particularly challenging if they had neurocognitive disorders such as those with a fetal alcohol spectrum disorder [52].

One of the main issues that most focus groups participants highlighted for research was the mental health of their young people. To help address this issue, participants suggested a youth centre could be built in their community. There were a number of facilities available for the community to use at the time of the study. These included a community pool which was opened only in the warmer months, a basketball court which was utilised for sports and regional competitions. Garnduwa was in charge of running the local football oval that was used regularly in football season as well as a recreational hall [113]. Occasionally there would be a movie night held in the hall for the children or a fun fair that came through the town. These events were however infrequent. Without a regular safe space for teenagers to go to after school, they are left wandering around the main town at night, depressed and vulnerable. The mental and emotional wellbeing of young Aboriginal people, particularly in extremely remote areas like the Fitzroy Valley is critical [114].

It was difficult to discuss suggestions for future research projects during the focus group discussions. In order to formulate research questions which addressed community priorities, more discussions would be needed with wider consultation conducted with the people of the Fitzroy Valley and Aboriginal community leaders.

### Strengths and weaknesses

This is the first project investigating preference for how consent is sought and evaluating the individual and community consent process for research with Aboriginal communities of the Fitzroy Valley. It provides a unique perspective to inform research methods. This project is scientifically rigorous, following standardised qualitative research methodology and designed in a way that is culturally respectful. This work is a collaboration of experienced researchers in partnership with locally respected Aboriginal leaders and Community Navigators employed to provide language and cultural guidance at each step of the project. It would be ideal if all focus groups were conducted and analysed in local language Conducted by Community Navigators. In this way, nuances of local language would not be lost through interpreting and the conversation between participants is likely to be more natural.

### Implications

There are very few published reports of researchers evaluating the consent process when working with Indigenous populations from around the world [3]. Because of the relative disadvantage of Indigenous people and entrenched racism in Australia [115] there is often a power differential between the researcher and the researched. The Picture Talk Project team identified key insights into how to overcome this and best conduct research in Indigenous communities. These include:

- Forming strong, trusting research relationships with Aboriginal leaders and their communities is essential. Seek the support of locally respected organisations.
- Acknowledging the skills and experience that are brought to the team by the Aboriginal research partners.
- Not assuming that communities will prioritise research agendas despite local events, such as cultural mourning practices.
- Employing locally respected Aboriginal people as Community Navigators to introduce you to Aboriginal communities and their leaders, interpret discussions in local language and seek consent for participation in research and evaluate whether consent is informed.
- Clarifying the distinction between research and health service.
- Participants expect to be paid for their time.
- Using pictures and stories to help explain research information. Having a logo for the project.
- Minimising text and jargon in the written consent form. Interpreting into Kriol when possible.
- Consulting the community on how to present the research information.

The Picture Talk Project is not aimed at creating a set of rules that are so difficult to uphold that this discourages researchers from working with Aboriginal communities. Aboriginal people understand that research can have value as it is important to ensure the survival, protection and well-being of the generations to come. The long-term aim of The Picture Talk Project is to provide guidance on planning research with Aboriginal communities in ways that go beyond tokenistic collaboration. With genuine partnerships, the project design will be enriched; and with community ownership of research outcomes, the intentions of the project will contribute to capacity building.

## Conclusion

When seeking consent for research, take into account local language, literacy and cultural protocols. This needs to be considered at each step of the research project [3–6, 83]. Current research guidelines are continuously revised with the aim to promote ethical research practices, however relationships need to be fostered and maintained with locally respected Aboriginal organisations who oversee and share ownership over the research project. Key community leaders should nominate locally respected Aboriginal people to be employed by the project as Community Navigators to interpret language and provide cultural guidance to visiting researchers when working with the community and individual participants. In this way the research is culturally informed at each step of the research process [83].
